# Association of *SELE* genotypes/haplotypes with sE-selectin levels in Taiwanese individuals: interactive effect of MMP9 level

**DOI:** 10.1186/1471-2350-13-115

**Published:** 2012-11-29

**Authors:** Semon Wu, Lung-An Hsu, Ming-Sheng Teng, Jeng-Feng Lin, Hsien-Hsun Chang, Yu-Chen Sun, Hsuan-Pu Chen, Yu-Lin Ko

**Affiliations:** 1 Department of Life Science, Chinese Culture University, Taipei, Taiwan; 2 Department of Medical Research, Buddhist Tzu Chi General Hospital, Taipei Branch, Taipei, Taiwan; 3 The First Cardiovascular Division, Department of Internal Medicine, Chang Gung Memorial Hospital, Taipei, Taiwan; 4 Chang Gung University College of Medicine, Taipei, Taiwan; 5 Division of Cardiology, Department of Internal Medicine, Buddhist Tzu Chi General Hospital, Taipei branch, 289 Jianguo Road, Xindian City, Taipei, 231, Taiwan; 6 Department of Laboratory Medicine, Chang Gung Memorial Hospital, Taipei, Taiwan; 7 Department of Neurology, University of California, San Francisco, USA; 8 School of Medicine, Tzu Chi University, Hualien, Taiwan

**Keywords:** E-selectin, Genetic association study, Polymorphism, Matrix metalloproteinase 9, Haplotype, Interaction

## Abstract

**Background:**

E-selectin is implicated in various inflammatory processes and related disorders. We aimed to investigate the role of *SELE*-gene genotypes/haplotypes on plasma levels of MMP9 and sE-selectin in Taiwanese individuals.

**Methods:**

Five hundred twenty individuals were enrolled. Seven tagging *SELE* single nucleotide polymorphisms were analyzed.

**Results:**

*SELE* genotypes were found associated with MMP9 and sE-selectin levels. Multivariate analysis identified that the most significant genetic polymorphism (rs5368 genotype) was independently associated with MMP9 levels (*P* < 0.001). One haplotype (GGAGAGT) was marginally associated with MMP9 levels (*P* = 0.0490). One *SELE* SNP, (rs3917406, *P* = 0.031) was associated with sE-selectin levels after adjusting for MMP9 and sICAM1 levels. Subgroup and interaction analysis revealed association of *SELE* SNP rs10800469 with sE-selectin levels only in the highest quartile of MMP9 level (*P* = 0.002, interaction *P* = 0.023). Haplotype analysis showed one haplotype (AAAAAGC) borderline associated with sE-selectin level (P = 0.0511).

**Conclusion:**

*SELE* genotypes/haplotypes are independently associated with MMP9 and E-selectin levels in Taiwanese individuals. The associations of *SELE* genotypes/haplotypes with sE-selectin levels are affected by MMP9 levels.

## Background

Chronic inflammation plays an import role in a variety of pathological disorders, including chronic pulmonary disease, chronic renal disease, and cardiovascular disease [1-3]. Chronic inflammation is also associated with abdominal obesity, smoking, and physical inactivity. The process of inflammation involves the interplay of many pro-inflammatory markers including C-reactive protein, fibrinogen, cytokines, adhesive molecules, and proteases [1,4]. E-selectin, an endothelial adhesive molecule, is a glycoprotein expressed exclusively on endothelial cells in response to inflammatory cytokines. It mediates the interaction of circulating leukocytes in various physiological and pathological settings [5]. E-selectin is also involved in activation of intracellular signaling pathways involved in the trans-endothelial migration of leukocytes [6]. Leukocyte and endothelial interactions contribute to a variety of vascular disease processes, such as acute and chronic inflammation and atherosclerosis. Soluble E-selectin (sE-selectin), thought to be shed from activated endothelial cells, has also been associated with atherosclerosis [7].

Single nucleotide polymorphisms (SNPs) of the *SELE* gene, encoding the E-selectin protein, have been linked to various disease states. The Ser128Arg gene variant, the most commonly reported SELE-gene polymorphism, is associated with a wide variety of disorders, including coronary artery disease, venous thrombosis, ischemic cerebral vascular disease, postoperative myocardial infarction, prognosis of colorectal cancer, restenosis after successful coronary angioplasty, severity of atherosclerotic arterial disease, peripheral arterial occlusive disease, and coronary calcification [8-12]. Common variants of the *SELE* gene have also been found associated with the susceptibility to either Graves’ disease or hypertension [13-15]. However, the association between *SELE* SNPs and E-selectin levels has been controversial [15-17], and the association between *SELE* SNPs and other inflammatory marker levels has not been reported. This investigation aimed to elucidate the association between *SELE* SNPs and the plasma levels of sE-selectin and MMP9, in Taiwanese individuals.

## Methods

### Study population

A total of 520 study participants were enrolled for study (mean ± SD): 264 men, age = 43.9 ± 9.4 years; 256 women, age = 45.8 ± 9.4 years. They responded to a questionnaire on their medical history and lifestyle characteristics, and were recruited during routine health examinations between October 2003 and September 2005 at the Chang Gung Memorial Hospital. All participants provided written informed consent. Exclusion criteria included cancer, current renal or liver disease, and a history of myocardial infarction, stroke, or transient ischemic attacks. In addition, individuals with a history of regular use of medication for diabetes mellitus, hypertension and/or lipid-lowering drugs were excluded from the analysis, because previous reports revealed that these agents affect the expression or concentrations of inflamatory markers [18-20]. The clinical and biometric features of the study group are summarized in Table 1. Hypertension was defined as a systolic blood pressure (BP) ≥ 140 mmHg and/or diastolic BP ≥ 90 mmHg, or regular use of antihypertensive medication. Obesity was defined as a body mass index (BMI) ≥ 25 kg/m^2^, according to the Asian criteria [21]. Current smokers were defined as individuals who smoked at least one cigarette per day at the time of the survey. The study was approved by the Ethics Committee of the Tzu-Chi Memorial Hospital.

**Table 1:** Baseline characteristics of the study subjects

	**Total**	**Men**	**Women**	***P *value**
Number	520	264	256	
Age (years)	44.8 ± 9.4	43.9 ± 9.4	45.8 ± 9.4	0.023
Systolic BP (mm Hg)	112.8 ± 16.1	113.8 ± 14.3	111.8 ± 17.8	0.172
Diastolic BP (mm Hg)	75.0 ± 10.0	77.0 ± 9.8	73.0 ± 9.9	< 0.001
Total cholesterol (mg/dL)	199.4 ± 36.2	202.7 ± 36.2	196.1 ± 36.0	0.036
HDL-cholesterol (mg/dL)	55.8 ± 14.5	49.9 ± 12.1	62.0 ± 14.1	< 0.001
LDL-cholesterol (mg/dL)	116.7 ± 33.1	120.5 ± 34.3	112.8 ± 31.5	0.008
Triglycerides (mg/dL)	139.0 ± 111.4	170.1 ± 137.6	107.0 ± 60.7	< 0.001
Body mass index (kg/m^2^)	24.1 ± 3.4	24.8 ± 3.1	23.4 ± 3.6	< 0.001
Waist circumference (cm)	84.5 ± 9.5	87.5 ± 7.6	81.4 ± 10.3	< 0.001
Current smokers (%)	19.8	35.2	3.9	< 0.001
Diabetes mellitus (%)	2.5	2.7	2.4	0.526
Glucose (AC) (mg/dL)	95.5 ± 22.3	97.9 ± 25.6	93.1 ± 17.9	0.014
HOMA-IR index	2.2 ± 1.4	2.3 ± 1.6	2.0 ± 1.1	0.002
CRP (mg/L)	1.0 ± 1.4	1.1 ± 1.4	1.0 ± 1.3	0.060
Fibrinogen (mg/dL)	260.2 ± 66.7	257.7 ± 68.6	262.8 ± 64.7	0.381
sE-selectin (ng/mL)	52.5 ± 25.7	59.8 ± 27.0	45.0 ± 22.0	< 0.001
sICAM1 (ng/mL)	239.8 ± 113.2	243.8 ± 109.6	235.8 ± 116.8	0.281
MMP9 (ng/mL)	141.9 ± 111.8	154.1 ± 112.8	129.4 ± 109.6	< 0.001

*BP*, blood pressure; *HDL*, high-density lipoprotein; *LDL*, low-density lipoprotein; *CRP*, C-reactive protein; *sICAM1*, soluble intercellular adhesive molecule 1; *sE-selectin*, soluble E-selectin; *MMP9*, matrix metalloproteinase 9; Continuous variables are presented as mean ± SD. CRP, *sICAM1*, sE-selectin and MMP9 values were transformed logarithmically before statistical testing to meet the assumption of normal distributions; however, the untransformed data are shown.

### Laboratory examination

Before starting the study, all participants underwent an initial screening assessment that included medical history, vital signs, a 12-lead electrocardiogram, and measurement of lipid variables and novel risk factors. A total of 15 mL of venous blood was collected in the morning after an overnight (8–12 hours) fast. Venous blood samples were collected from an antecubital vein using a 21-gauge needle. Serum, EDTA, sodium fluoride, and sodium citrate plasma were obtained by centrifugation at 3000 × g for 15 minutes at 4°C. Immediately thereafter, serum/plasma samples were frozen and stored at −80°C prior to analysis. All measurements were performed in a central laboratory. The homeostasis model assessment of insulin resistance (HOMA-IR) index was calculated using the formula: HOMA-IR = fasting serum insulin (μU/mL) × fasting plasma glucose (mmol/L)/22.5.

### Assays

Most markers, including C-reactive protein (CRP), sE-selectin, sICAM-1 and MMP9 were measured using a sandwich enzyme-linked immunosorbent assay (ELISA) developed in-house. All in-house kits were compared with commercially available ELISA kits and showed good to excellent correlation [22-24]. Serum insulin levels were measured using an immunoradiometric assay (Bio-source, Nivelles, Belgium). Glucose was determined enzymatically using the hexokinase method. Total cholesterol and triglyceride concentrations were measured by automatic enzymatic colorimetry. High density lipoprotein (HDL) cholesterol levels were measured enzymatically after phosphotungsten/magnesium precipitation. Low density lipoprotein (LDL) cholesterol was either calculated from the Friedewald formula or, in patients with triglycerides > 400 mg/dL, detected with commercial reagents by standard protocol. Plasma fibrinogen levels were determined using the Clauss method adapted for a Sysmex CA1-1500 instrument in the Clinical Hematology Laboratory.

### Genomic DNA extraction and genotyping

Genomic DNA was extracted as reported previously [25,26]. From the published sequence of the *SELE* gene, oligonucleotide primers were generated to amplify fragments of genomic DNA containing the genetic polymorphisms reported on the websites of GenePipe (http://genepipe.ngc.sinica.edu.tw/visualsnp) and the NCBI SNP database (http://www.ncbi.nlm.nih.gov/SNP). Seven SNPs within *SELE* were chosen in this study (Additional file 1: Table S1). Six tagSNPs were chosen according to a previous study reported by Chen et al. [15], who selected tagSNPs by running the tagger program implemented in Haploview software. The SNP rs5361 (Ser128Arg) was selected because it was located in an exon, resulted in an amino-acid substitution and has been linked to a wide variety of disorders [8-12]. Genotyping for the SNPs rs5368, rs3917412, and rs5361 was performed using polymerase chain reaction and restriction enzyme digestion. Genotyping for the SNPs rs10800469, rs3917406, rs2179172, and rs3917419 were performed using TaqMan SNP Genotyping Assays obtained from Applied Biosystems (ABI, Foster City, CA, USA).

### Statistical analysis

To determine the association between gender and other clinical data, we used chi-square test and two-sided *t*-test for categorical variables and continuous variables, respectively. The clinical characteristics that were continuous variables are expressed as means ± SDs and were tested using a two-sided *t*-test or analysis of variance (ANOVA). Furthermore, a general linear model was applied to capture the major effect of each polymorphism on clinical phenotype variables, with BMI, age, gender and smoking status as confounding covariates. We used recessive models for numeric association test after recoding our SNPs from categorical variables to continuous variables, such as 0, 1 of a particular allele. CRP, sICAM1, sE-selectin, sP-selectin, and MMP9 values were transformed logarithmically before analysis to adhere to an assumption of normality. The result was adjusted by false discovery rate (FDR) for multiple test correction and the regression coefficient with FDR *P* value <0.05 was considered as significant. Besides, stepwise linear regression analysis was used to determine independent predictors of MMP9 levels. Interactions between each SNP, the level of sE-selectin, and MMP9 levels were tested with two-way ANOVA. When interaction terms were found to be significant, stratified analyses of the genetic variants of the genotypes (e.g., target genotypes affected by MMP9 levels) and sE-selectin level were performed to further investigate interactive effects while controlling for other variables including age, gender, BMI, and smoking. All calculations were performed with standard statistical SPSS software. For the assessment of association between haplotypes and clinical variables, Golden Helix SVS Win32 7.3.1 software were applied and the haplotype with FDR *P* value <0.05 was reported. We used SNPStats software (available at http://bioinfo.iconcologia.net/SNPstats) [27] to calculate the linkage disequilibrium between SNPs and the deviation from Hardy-Weinberg equilibrium. Values of *P* < 0.05 using a two-sided test were considered statistically significant.

## Results

### Clinical and biochemical characteristics

A summary of demographic features, clinical and lipid profiles, and inflammatory biomarkers for the study participants stratified by gender is provided in Table 1. No significant deviations from the Hardy-Weinberg equilibrium were detected for the studied polymorphisms (*P* = 0.73, 0.71, 0.17, 0.21, 0.88, 0.92, and 0.80 for SNPs rs10800469, rs3917406, rs5361, rs3917412, rs2179172, rs3917419, and rs5368, respectively). All these SNPs were in strong pairwise linkage disequilibrium, except for rs5361, for which the calculation was not possible due to the identification of only two of this genotype among the study participants (Additional file 1: Table S2).

### Associations of the SELE gene polymorphisms with serum levels of MMP9 levels

Table 2 presents a comparison of the distributions of the serum MMP9 concentrations according to *SELE* genotype status. With the recessive model, the minor alleles of rs3917419 and rs5368 were found to be associated with higher MMP9 levels (*P* = 0.002 and *P* = 1.4 × 10^-4^, respectively) after adjusting for age, gender, smoking status, and BMI (Table 2). These associations remained statistically significant after multiple comparisons adjustment (FDR *P* = 0.005 and *P* = 6.9 × 10^-4^, respectively).

**Table 2:** Serum MMP9 and sE-selectin levels by *SELE *genotype in recessive model

**rs Number and Genotypes**		**MMP9**	***P *value**^**#**^	**Adjusted**	**sE-selectin (mg/L)**	***P *value**^**#**^	*P *value*	**Adjusted**
		**(mg/L)**		***P *value**				***P *value**
rs10800469	AA (n = 113)	143.0 ± 107.4	0.929	1	55.0 ± 30.2	0.162	0.060	0.180
	AG + GG (n =388)	141.9 ± 113.9			51.9 ± 24.3			
rs3917406	GG (n = 122)	159.4 ± 138.9	0.066	0.131	49.0 ± 27.6	0.023	0.005	0.031
	AA + AG (n = 378)	136.6 ± 102.0			53.7 ± 25.1			
rs5361	AA (n = 487)	142.9 ± 113.4	0.477**	-	52.6 ± 25.7	0.943**	0.865**	-
	AC (n = 12)	118.1 ± 62.7			56.3 ± 31.3			
	CC (n = 0)	-			-			
rs3917412	AA (n = 40)	139.4 ± 84.5	0.964	0.963	49.5 ± 19.7	0.624	0.824	0.824
	AG + GG (n = 459)	142.6 ± 114.7			52.9 ± 26.3			
rs2179172	CC (n = 21)	137.8 ± 100.5	0.872	1	50.1 ± 24.9	0.772	0.492	0.590
	AA + AC (n = 476)	143.0 ± 113.1			52.9 ± 25.9			
rs3917419	AA (n = 15)	209.5 ± 119.3	0.002	0.005	61.1 ± 47.2	0.326	0.224	0.336
	AG + GG (n = 485)	139.4 ± 110.9			52.4 ± 24.8			
rs5368	TT (n = 25)	234.0 ± 209.9	1.1 × 10^-4^	6.9 × 10^-4^	45.6 ± 14.4	0.354	0.181	0.336
	CC + CT (n = 476)	137.0 ± 102.5			52.6 ± 25.1			

Continuous variables are presented as mean ± SD. MMP9 and sE-selectin values were logarithmically transformed before statistical testing to meet the assumption of normal distributions; however, the untransformed data are shown.

^#^Multiple linear regression, adjusted for age, gender, smoking and BMI. *Multiple linear regression, adjusted for age, gender, smoking, BMI, sICAM1 and MMP9 levels. Adjusted *P* values were computed by the false discovery rate method. **Comparison between *AA* and *AC* genotypes.

### SELE haplotypes and MMP9 levels

Because the single SNP regression demonstrated that multiple sites within the *SELE* gene significantly affect MMP9 level, haplotypes were inferred to capture possible allelic associations. In the present investigation, seven common haplotypes (≥ 1% frequency) were derived from seven SNPs, accounting for 99.6% of all inferred haplotypes (Table 3). In haplotype analysis, two haplotypes inferred from seven SNPs (AAAAAGC and GGAGAGT) were found to be associated with either decreased or increased MMP9 levels (*P* = 0.0234 and *P* = 0.007, respectively). However, only the observed association between haplotype GGAGAGT and MMP9 levels remained marginally significant after multiple comparisons adjustment (FDR *P* = 0.0490).

**Table 3:** Association of *SELE *locus haplotypes with MMP9 level (adjusted for age, gender, BMI, and smoking)

	**SNP1**	**SNP 2**	**SNP 3**	**SNP 4**	**SNP 5**	**SNP 6**	**SNP 7**	**Frequency**	**Coef**	**SE**	***P *value**	***Adjusted P *value**
H1	A	A	A	A	A	G	C	0.2542	-0.0514	0.0227	0.0234	0.0819
H2	G	G	A	G	A	G	T	0.2266	0.0512	0.0190	0.0070	0.0490
H3	G	G	A	G	C	G	C	0.2080	-0.0112	0.0200	0.5746	0.8044
H4	A	A	A	G	A	A	C	0.1384	0.0144	0.0221	0.5144	0.9002
H5	G	A	A	G	A	G	C	0.0651	-0.0086	0.0310	0.7822	0.7822
H6	A	A	A	G	A	G	C	0.0592	-0.0261	0.0352	0.4572	1
H7	G	G	A	G	A	G	C	0.0484	-0.0167	0.0370	0.6524	0.7611

*SNP1*: rs10800469, *SNP2*: rs3917406, *SNP3*: rs5361, *SNP4*: rs3917412, *SNP5*: rs2179172, *SNP6*: rs3917419, *SNP7*:rs5368. Multiple linear regression, adjusted for age, gender, smoking and BMI. Adjusted *P* values were computed by the false discovery rate method.

### Stepwise regression analysis of MMP9 levels using a general linear model in the study population

In addition to five previously reported independent variables—age, current smoker, fibrinogen levels, fasting plasma glucose level, and an *MMP9* SNP rs2274756 [28]—the *E-selectin* gene variants were used for further multivariate analysis. In a stepwise regression analysis, the most significant genetic polymorphism rs5368 genotypes with recessive model, age, gender, smoking status, and fibrinogen levels were all independently associated with MMP9 levels (*P* < 0.001) (Table 4).

**Table 4:** MMP9 levels: stepwise linear regression analysis, including genotype

**Variable**	**Beta**	***R***^**2*a***^	***P *value**
Current smoker	0.165	0.054	< 0.001
rs5368 TT genotype	0.168	0.083	< 0.001
rs2274756 AA genotype	0.110	0.098	0.009
Fibrinogen	0.119	0.108	0.006
Age	-0.124	0.118	0.004
Gender	-0.090	0.127	0.029

^*a*^Cumulative R^2^. Multiple linear regression, adjusted for age, gender, smoking status, BMI, fibrinogen, fasting plasma glucose, and MMP9 rs2274756 genotype.

### Associations of the SELE genotypes/haplotypes with sE-selectin levels: focus on interactive effects

**Figure 1:**
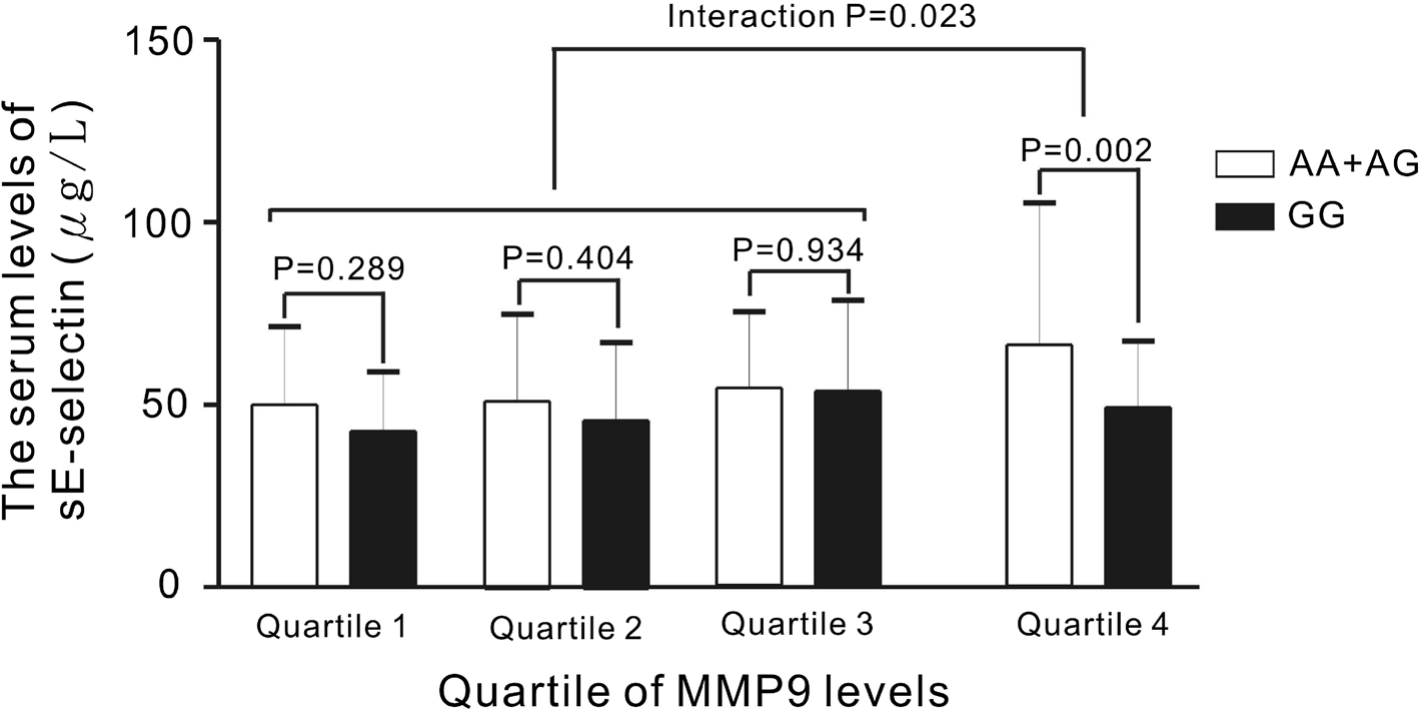
**Interactive effect of MMP9 levels on the association between rs10800469 and sE-selectin levels.** After adjusting for clinical covariates, minor allele of rs10800469 of the *SELE* gene was found to be associated with decreased sE-selectin levels, predominantly in highest quartile of MMP9 levels subjects (*P* = 0.002). Interaction analysis revealed an interaction of MMP9 levels with the rs10800469 genotype (interaction P = 0.023, after the adjustment of age, gender, BMI, smoking, sICAM1 and MMP9).

In contrast to the highly significant association with MMP9 levels, after the adjustment for age, gender, smoking status, and BMI, there was a marginally significant association of the *SELE* gene rs3917406 polymorphism with sE-selectin level (*P* = 0.023) (Table 2). Because of the significant association of sE-selectin levels with sICAM1, and MMP9 levels (r = 0.306, *P* < 0.001 for sICAM1 and r = 0.177, *P* < 0.001 for MMP9, respectively), we further elucidated possible interactive effects of sICAM1 and MMP9 levels on the association between the *SELE* gene polymorphisms and sE-selectin level. By multivariate analysis with sICAM1 and MMP9 levels included for analysis, significant associations of rs3917406 was found with sE-selectin level (*P* = 0.005) (Table 2). The association also remained statistically significant after multiple comparisons adjustment (FDR *P* = 0.031) (Table 2). Subgroup analysis revealed association of *SELE* SNP rs10800469 with sE-selectin only in the highest quartile of MMP9 levels (66.0 ± 38.7 *vs.* 48.0 ± 18.4 mg/dL, AA + AG *vs.* GG genotypes, *P* = 0.002). Interaction analysis revealed interaction of *SELE* genotypes and MMP9 levels on sE-selectin levels (interaction *P* = 0.023) (Figure 1). Haplotype analysis showed two haplotypes, AAAAAGC and GGAGCGC, were associated with sE-selectin levels (*P* = 0.0073 and *P* = 0.0425, respectively) (Table 5). However, after multiple comparisons adjustment, the association of GGAGCGC became insignificant; whereas the apparent association between haplotype AAAAAGC and sE-selectin levels became borderline significant (FDR *P* = 0.0511).

**Table 5:** Association of *SELE *locus haplotypes with sE-selectin level (adjusted for age, gender, BMI, smoking, sICAM1 and MMP9)

	**SNP 1**	**SNP 2**	**SNP 3**	**SNP 4**	**SNP 5**	**SNP 6**	**SNP 7**	**Frequency**	**Coef**	**SE**	***P *value**	***Adjusted P *value**
H1	A	A	A	A	A	G	C	0.2542	0.0421	0.0157	0.0073	0.0511
H2	G	G	A	G	A	G	T	0.2266	-0.0167	0.0133	0.2072	0.3626
H3	G	G	A	G	C	G	C	0.2080	-0.0281	0.0139	0.0425	0.1487
H4	A	A	A	G	A	A	C	0.1384	0.0255	0.0152	0.0935	0.2182
H5	G	A	A	G	A	G	C	0.0651	-0.0175	0.0213	0.4124	0.5774
H6	A	A	A	G	A	G	C	0.0592	0.0070	0.0242	0.7725	0.9012
H7	G	G	A	G	A	G	C	0.0484	-0.0066	0.0256	0.7968	0.7968

*SNP1*: rs10800469, *SNP2*: rs3917406, *SNP3*: rs5361, *SNP4*: rs3917412, *SNP5*: rs2179172, *SNP6*: rs3917419, *SNP7*:rs5368. Multiple linear regression, adjusted for age, gender, smoking, BMI, sICAM1 and MMP9 levels. Adjusted *P* values were computed by the false discovery rate method.

## Discussion

The present investigation analyzed the association of genetic variants of the *SELE* gene with serum inflammatory marker levels. The results showed that, in addition to previously reported *MMP9* genotypes, the *SELE* gene is the second locus that is significantly associated with MMP9 level in Taiwanese individuals. The association of *SELE* genotypes/haplotypes with MMP9 level is significant after the adjustment of other independent factors including age, gender, smoking status, fibrinogen levels, and *MMP9* genotypes. To the best of our knowledge, this is the first investigation revealing that the genetic determinants of MMP9 levels may include a cellular adhesive molecule locus. Further, *SELE* genotypes/haplotypes are independently associated with sE-selectin levels, especially after the adjustment of sICAM1 and MMP9 levels. Interaction analysis also revealed an interactive effect of MMP9 level on the association of *SELE* genotypes with sE-selectin level. These results provide further evidence of the close relationship between cellular adhesive molecules and matrix metalloproteinases.

The association between cellular adhesive molecules and matrix metalloproteinases has been reported previously. Aoudjit et al. [29] demonstrated that firm adhesion of T lymphoma cells to endothelial cells participates in the production of MMP9 in both cell types through bi-directional signaling pathways, and identified ICAM-1/LFA-1 as a key interaction in the up-regulation of MMP9 in T lymphoma cells. MMP9 also cleaved membrane ICAM1 in an in vitro model, which may result in increased sICAM1 [30]. Although direct evidence linking E-selectin and MMP9 molecules is absent, there are several possibilities for the involvement of the *SELE* gene in the signaling pathways affecting gene expression and the serum level of MMP9. First, both E-selectin and MMP9 have been shown to be involved in the process of trans-endothelial migration (TEM) of leukocytes and cancer cells. Endothelial E-selectin is quickly and transiently expressed following a challenge by proinflammatory stimuli and plays a pivotal role in mediating cell-cell interactions between breast cancer cells, colon carcinoma cells, leukocytes, and endothelial cells [31-34]. Historical literature reporting on MMP9 function in vitro also provided a compelling story implicating MMP9 as a rate-limiting extra-cellular protease involved in cell migration across basement membranes, a process that has been found to be orchestrated by an ever-increasing number of molecules, including selectins, integrins, and their ligands on the endothelium that mediate rolling, firm adhesion, and diapedesis [35-37]. Second, studies on endothelial signaling by adhesive molecules and the concomitant analysis of associated adapter proteins have been most successful for E-selectin and ICAM-1. There is ample evidence for the signal capacity of E-selectin, both towards the actin cytoskeleton as well as to p42 MAPK/ERK activation and the induction of c-fos [38-40]. MMP9, an inducible enzyme, can be expressed in a number of cells under the action of TNF-α, IL-1β, PDGF, and some other growth factors. This expression has been shown to be mediated via ERK1/2, p38 MAPK, and/or JNK signal transduction pathways [41-43]. Third, both E-selectin and MMP9 have been shown to be involved in cancer-cell invasion and metastasis. For a long time, the proteolytic remodeling of extracellular matrix by MMPs, serine proteases, and cathepsins was considered to be a critical determinant of tumor cell invasiveness [44]. Thus, further studies involving direct analysis of the interaction between E-selectin and MMP9 molecules using in vitro models may help us to elucidate the role of E-selectin on MMP9 expression.

Previous studies on the association between *SELE* SNPs and sE-selectin levels have been controversial. Chen et al. [15] revealed significant association of *SELE* haplotypes with sE-selectin level in a Chinese population. In a genome-wide association study, Paterson et al. [17] found no evidence (probability value < 0.01) of association between SNPs in or near the *SELE* gene and sE-selectin levels. After analyzing 628 individuals from different ethnic populations, Miller et al. [16] found no evidence of association between the *SELE* S128R polymorphism and circulating sE-selectin levels. Our data revealed an association between *SELE* genotypes and sE-selectin levels only in the highest quartile of MMP9 levels. Proteolytic cleavage of cellular adhesive molecules on the cell membrane by proteases has been reported before, however, it is unknown if the membrane E-selectin molecule can be cleaved by MMP9. Significant associations between sE-selectin and MMP9 levels were noted in our study, suggesting a possible interaction between these two molecules. Further study is necessary to elucidate the role of MMP9 on sE-selectin level.

Some limitations of our study deserve consideration. One limitation of the study is the relatively low number of subjects genotyped; replication in a second cohort would improve the strength of the study. Furthermore, only seven of the *SELE* SNPs were analyzed, and the incomplete genotyping may not represent all of the haplotypes in the *SELE* gene. Another limitation of this study is its cross-sectional design, which could only draw limited inferences with regard to the relationships between exposure and outcome. After the use of FDR for multiple testing corrections, only few *SELE* genetic variances remained significantly associated with MMP9 or sE-selectin level and the association between haplotypes on MMP9 or sE-selectin levels became marginal. Therefore, cautious and conservative interpretation of the data is needed. Independent association studies with larger sample sizes and more complete genotyping, especially using a prospective design, are needed to confirm these results before any definitive conclusions can be drawn.

## Conclusions

In conclusion, our data revealed for the first time that *SELE* SNPs are independently associated with MMP9 levels, in addition, *SELE* gene variants interacted with MMP9 levels for the association with sE-selectin levels in Taiwanese individuals. The findings have implications for the prediction of inflammation-related disorders. Further studies of the interrelationship between cellular adhesive molecules and matrix metalloproteinases are needed to confirm MMP9 levels as a possible link between *SELE* genotypes/haplotypes and inflammation-related disorders.

## Abbreviations

SNPs: Single nucleotide polymorphisms; BMI: Body mass index; BP: Blood pressure; CRP: C-reactive protein; HDL: High-density lipoprotein; LDL: Low-density lipoprotein; sICAM1: Soluble intercellular adhesive molecule 1; sE-selectin: Soluble E-selectin; MMP9: Matrix metalloproteinase 9; TEM: Trans-endothelial migration.

## Competing interests

The authors declare that they have no competing interests.

## Authors’ contributions

S W and L-A H participated in genotyping, performed statistical analysis and drafted the manuscript. M-S T and H-H C prepared the DNA samples and participated in genotyping. J-F L participated in sample collection and prepared the DNA samples. Y-C S participated in ELISA assay. H-P C performed and corrected statistical analysis. Y-L K supervised the study and revised the manuscript. All authors read and approved the final manuscript.

## Pre-publication history

The pre-publication history for this paper can be accessed here:

http://www.biomedcentral.com/1471-2350/13/115/prepub

## Supplementary Material

Additional file 1 **Table S1.** Primer sequences and restriction enzyme (RE) used in SELE gene polymorphisms. **S2**. Linkage disequilibrium between *SELE* genetic polymorphisms. Click here for file
