# Supplementary material for: Association of SELE genotypes/haplotypes with sE-selectin levels in Taiwanese individuals: interactive effect of MMP9 level
Source: BMC Med Genet. 2012 Nov 29;13:115. doi: 10.1186/1471-2350-13-115 (PMC3532335; doi:10.1186/1471-2350-13-115)
Supplement: Additional file 1 — Table S1. Primer sequences and restriction enzyme (RE) used in SELE gene polymorphisms. S2. Linkage disequilibrium between SELE genetic polymorphisms. [file 1471-2350-13-115-S1.doc]

Supplement Table 1. Primer sequences and restriction enzyme (RE) used in *SELE* gene polymorphisms.

| SNP number | Primer sequence | PCR size  and RE | Allele | Minor allele  (frequency) | Amino acid | Location |
| --- | --- | --- | --- | --- | --- | --- |
| rs10800469 | TaqMan SNP Genotyping Assays |  | A/G | A (0.46) |  | Promoter |
| rs3917406 | TaqMan SNP Genotyping Assays |  | A/G | G (0.48) |  | Intron |
| rs5361 | (F)5'-GATAGGTGGGCCTGAAACTCAG-3'  (R)5'-CACTTGAGTCCACTGAAGCCAG-3' | 272 bp  Bts I | A/C | C (0.01) | R/S | Exon4 |
| rs3917412 | (F)5'-GCACACAGGACACATTCAATAA-3'  (R)5'-AACACAGCTATGGAACATTTCC-3' | 314 bp  Tsp45 I | A/G | A (0.30) |  | Intron |
| rs2179172 | TaqMan SNP Genotyping Assays |  | A/C | C (0.20) |  | Intron |
| rs3917419 | TaqMan SNP Genotyping Assays |  | A/G | A (0.14) |  | Intron |
| rs5368 | (F)5'-GAAGAGCTTGGGAACTTGGTTA-3’ (R)5'-TCAATTCTACCTTGGCAGGAAG-3’ | 284 bp  Nla III | C/T | T (0.22) | Y/H | Exon9 |

Supplement Table 2. Linkage disequilibrium between *SELE* genetic polymorphisms

|  | rs10800469 | rs3917406 | rs3917412 | rs2179172 | rs3917419 | rs5368 |
| --- | --- | --- | --- | --- | --- | --- |
| rs10800469 | - | 0.7402 | 0.4942 | 0.2151 | 0.1836 | 0.2458 |
| rs3917406 | - | - | 0.3865 | 0.2760 | 0.1520 | 0.3087 |
| rs3917412 | - | - | - | 0.1079 | 0.0696 | 0.1224 |
| rs2179172 | - | - | - | - | 0.0419 | 0.0668 |
| rs3917419 | - | - | - | - | - | 0.0463 |
| rs5368 | - | - | - | - | - | - |

The values represent r^2^
